# Extension of host lifespan by polyamines derived from gut bacteria

**DOI:** 10.1128/mbio.01484-26

**Published:** 2026-09-10

**Authors:** Hiromi Shimokawa, Emil Salim, Aki Hori, Tsuyoshi Fujita, Hayato Takihara, Hibiki Kadoguchi, Yuki Kontani, Rika Hirano, Mitsuharu Matsumoto, Shujiro Okuda, Takayuki Kuraishi, Shin Kurihara

**Affiliations:** 1 Research Institute for Bioresources and Biotechnology, Ishikawa Prefectural University, Suematsu 73985, Nonoichi, Ishikawa, Japan; 2 Faculty of Biology-Oriented Science and Technology, Kindai University 74014 , Kinokawa, Wakayama, Japan; 3 Department of Faculty of Pharmacy, Institute of Medical, Pharmaceutical and Health Sciences, Kanazawa University, Kakuma-machi 12858, Kanazawa, Ishikawa, Japan; 4 Medical AI Center, Niigata University School of Medicine 196869, Niigata, Niigata, Japan; 5 Dairy Science and Technology Institute, Kyodo Milk Industry Co., Ltd., Hirai, Hinode-machi 476862 , Nishitama-gun, Tokyo, Japan; Georgia Institute of Technology, Atlanta, Georgia, USA

**Keywords:** genetically engineered bacteria, gnotobiotic, lifespan, metabolite, polyamine

## Abstract

Metabolites produced by gut bacteria are taken up by the host and have a direct impact on its health. However, to our knowledge, no studies have investigated the effects of gut bacterial metabolites on the lifespan of the host using gnotobiotic animals colonized with gut bacteria deficient in biosynthetic genes involved in the production of specific metabolites. Polyamines, such as putrescine and spermidine, are among the most important metabolites of gut bacteria. Previous studies have shown that increasing polyamines in the colon of mice extends their lifespan and biological functions. In this study, we produced gnotobiotic flies colonized with *Escherichia coli*, in which polyamine biosynthetic genes were deleted or complemented. Gnotobiotic flies were fed a polyamine-free diet, and the effects of metabolites derived solely from gut bacteria on the lifespan of the host were analyzed. The results showed that polyamines derived from gut bacteria significantly prolonged the lifespan of the flies. Furthermore, the expression of *TotM* and *Halo* was suppressed in flies monocolonized with polyamine-producing *E. coli* compared with those monocolonized with mutant bacteria incapable of producing polyamines.

**IMPORTANCE:**

The intestinal tract of a human contains more than 150 species of gut bacteria, which comprise the gut microbiota. Only a very small number of gut bacteria, compared to the bacteria in feces, can come into contact with human tissues due to the barrier mechanism of the intestinal tract, but metabolites of the gut microbiota can pass through the intestinal epithelium and be taken into the body, directly affecting the health of the host. Thus, the importance of gut bacterial metabolites has received increasing attention in recent years. However, analysis into the effects of gut bacterial metabolites on host animals has been limited to only part of the host animal life cycle. In this study, we used *Drosophila melanogaster*, which has a short average lifespan, as a host and measured its lifespan to provide a simple analysis of the lifetime effects of gut bacterial metabolites on the host.

## INTRODUCTION

In recent years, it has been reported that gut bacteria affect host health conditions, such as obesity (1), diabetes (2), autism (3), depression (4), and athletic performance (5); moreover, it has been suggested that specific gut bacteria or microbiota exert these effects. Previous flow cytometry analyses reported bacterial densities of 2.3 × 10^10^ cells/g in feces (6), 2.2 × 10^9^ cells/mL in the intestinal epithelial mucus layer (7), and 1 × 10^3^ cells/mL in the blood of septic patients (8). These findings indicate that gut bacterial cells are abundant in the intestinal lumen and mucus-associated compartments, whereas their translocation into the internal body compartment, especially the bloodstream, is limited by the intestinal barrier system, including the mucus layer, epithelial barrier, and mucosal immune factors. In contrast, many gut bacterial metabolites can be absorbed across the intestinal epithelium, enter the systemic circulation, and influence host physiology (9, 10). Therefore, the effects of gut bacteria on host health may depend largely on bacterial metabolites rather than on the direct systemic dissemination of bacterial cells (11).

A type of important bacterial metabolite is polyamines, which consist of aliphatic hydrocarbons containing two or more amino groups. The major polyamines are putrescine, spermidine, and spermine. The concentrations of putrescine, spermidine, and spermine in the feces of SPF mice (12) were approximately 40, 300, and 40 μM, respectively, while the concentrations of putrescine and spermidine in the feces of healthy humans (13) were approximately 800 and 50 μM, respectively. The above polyamine concentrations in feces are considered to be equivalent to those in the colonic lumen. The polyamine concentration in the colon tissue of gnotobiotic mice, in which polyamine-producing *Escherichia coli* were mono-colonized in the intestinal tract, was statistically significantly higher by approximately 1.4–2 times compared to that in mice mono-colonized with polyamine-nonproducing *E. coli* (12). This suggests that polyamines in the intestinal lumen are actively taken up by the host into the colonic tissue. In addition to gut bacteria, diet is an important source of polyamines in animals, which also synthesize polyamines by themselves (14). The concentrations of spermine and spermidine in the intestinal tissue of SPF mice are approximately 7 and 3 nmol/mg protein at 3 weeks of age and 6 and 3 nmol/mg at 10 weeks of age, respectively (15). It is hypothesized that these concentrations are the result of a combination of polyamines synthesized by gut bacteria and absorbed into the tissue via the colonic epithelium, polyamines derived from the diet, and polyamines biosynthesized by the host.

Recently, various health benefits of increased polyamines in the colon, which were derived from gut bacteria, have been reviewed (14). For instance, oral administration of *Bifidobacterium animalis* increased polyamine concentration in the colon and prolonged the lifespan of mice (16). The simultaneous oral administration of arginine, a precursor of putrescine, with *B. animalis* also increased polyamine concentration in the colon and enhanced the memory of mice (17). However, based on the experimental design of these studies, lifespan extension might be a side effect of administering *B. animalis* and arginine to the host. In addition, as the experimental animals in the above study were fed diets containing polyamines (16), it is not possible to conclude that the health-promoting effects associated with increased polyamine concentration in the colon were due to polyamines derived from gut bacteria.

Diet is an important source of polyamines for animals, and several health-promoting effects of oral intake of polyamines have been reported (18, 19), such as improved cardiac function in aged mice fed spermidine (18) and improved memory in flies fed spermidine (20). Furthermore, oral administration of polyamines to animals (mice and flies) has been reported to prolong their lifespan (19, 21, 22). Although all these studies used animals with gut microbiota capable of producing polyamines, it was not clearly shown that the beneficial effects of polyamines on host health were derived solely from the dietary polyamines. Overall, previous studies (19, 23) have reported various important health-promoting effects in animals without distinguishing between the two sources of polyamines: diet and gut bacteria.

Recently, by using gnotobiotic mice monocolonized with genetically engineered *Escherichia coli* lacking the ability to produce polyamines, our research group has demonstrated that polyamines derived from gut bacteria contributed to the protection of the intestinal mucosa via eIF5A hypusination (12). However, long-term experiments are required to analyze the lifespan of mice because the lifespan of mice is relatively long (2 years); moreover, removal of polyamines from the mice’s diet is difficult. *Drosophila melanogaster* can grow on a polyamine-free diet (24) and has a short lifespan of approximately 2 months, which makes this species of fly suitable for lifespan analysis. Therefore, in the present study, we monocolonized germ-free *D. melanogaster* with wild-type or genetically engineered *E. coli* strains, in which polyamine biosynthetic genes were deleted or complemented, and fed the flies a polyamine-free diet to demonstrate that polyamines derived from gut bacteria contribute to prolonging the host lifespan.

## RESULTS

### Lifespan-extending effects of exogenous polyamines exclusively derived from diet

**Fig 1:**
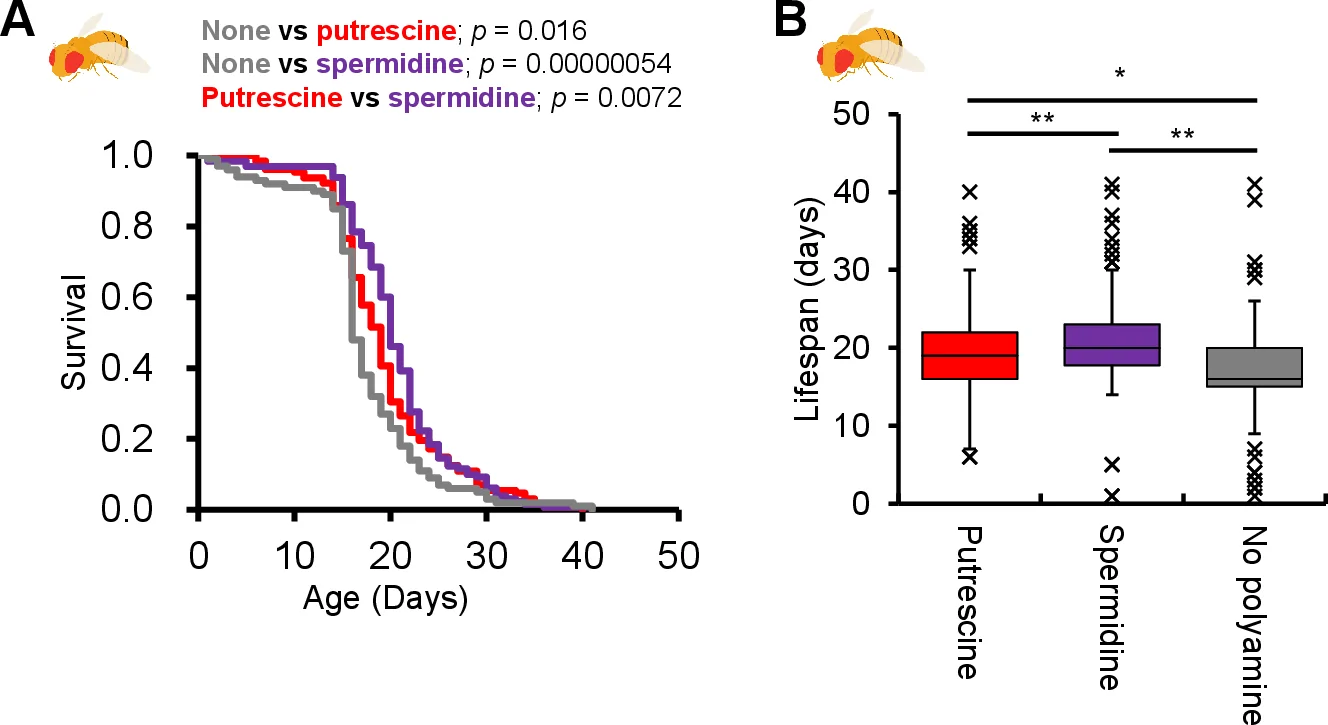
Effect of dietary polyamines on lifespan extension. (**A**) Survival curve of germ-free flies fed chemically defined holidic diets supplemented with polyamines. The red, purple, and gray curves show the survival curves of the group of flies grown on holidic diet supplemented with putrescine at a final concentration of 15 mM (Table S3), spermidine at a final concentration of 10 mM (Table S4), and the holidic diet without polyamines (Table S2), respectively. Five containers were utilized for the rearing of 27, 26, 23, 27, and 25 flies, respectively, yielding a total of 128 flies in the putrescine group. Five containers were utilized for the rearing of 24, 25, 27, 24, and 30 flies, respectively, yielding a total of 130 flies in the spermidine group. Five containers were utilized for the rearing of 16, 15, 21, 28, and 20 flies, respectively, yielding a total of 100 flies in the no polyamine group. The number of dead flies in each container was summed daily, and the survival rate was calculated. Differences in the shapes of the survival curves were analyzed using the generalized Wilcoxon test. (**B**) The lifespan of each group is shown as a boxplot. Whiskers are minimum and maximum values within the 1.5 × interquartile range (IQR), and the crosses are extreme outliers outside the 1.5× IQR. The horizontal line within each box indicates the median life expectancy. Differences in median life expectancy were tested using the Mann–Whitney *U* test, and differences in average lifespan were tested using the Steel–Dwass test. The red, purple, and gray bars show the average lifespan of the group of flies grown on a diet supplemented with putrescine, with spermidine, and without polyamines, respectively. **, *P* < 0.01; *, *P* < 0.05.

First, the effects of diet-derived polyamines on the lifespan of flies were analyzed. Since the existing diet of flies contains polyamines (Fig. S1), we used a polyamine-free chemically defined diet (holidic diet [24] [Table S2]) in the present study. Germ-free flies were raised on holidic diets supplemented with putrescine (Table S3) or spermidine (Table S4) at final concentrations of 15 mM and 10 mM, respectively, and without polyamines (Table S1). Survival curves were generated for each group of flies consuming each diet, and the shapes of survival curves were compared using the generalized Wilcoxon test. The survival curves were significantly different between the groups of flies fed putrescine- or spermidine-supplemented diets and those fed the polyamine-free diet (*P =* 0.016 and *P =* 5.4 × 10^−7^, respectively; Fig. 1A). The survival curves also differed between the putrescine- and spermidine-supplemented groups (*P* = 0.0072; Fig. 1A). Furthermore, the median life expectancy was 20 days in both the putrescine- and spermidine-supplemented groups, and each group showed a significantly higher median lifespan than the polyamine-free group (16 days) according to the Mann–Whitney *U* test (putrescine vs. no polyamine, *P* = 0.0044; spermidine vs. no polyamine, *P* = 0.00052; Fig. 1B). In addition, the average lifespan was 20.8, 21.1, and 18.2 days in the groups fed putrescine-supplemented, spermidine-supplemented, and polyamine-free diets, respectively. These average lifespans were significantly different between the groups of flies fed the polyamine-free diet and those fed putrescine- or spermidine-supplemented diets according to the Steel–Dwass method (*P =* 0.012 and *P =* 0.0015, respectively). These results indicate that diet-derived polyamines are sufficiently effective in extending the lifespan of germ-free flies, which cannot obtain polyamines derived from gut bacteria.

### Establishment of the gnotobiotic flies

**Fig 2:**
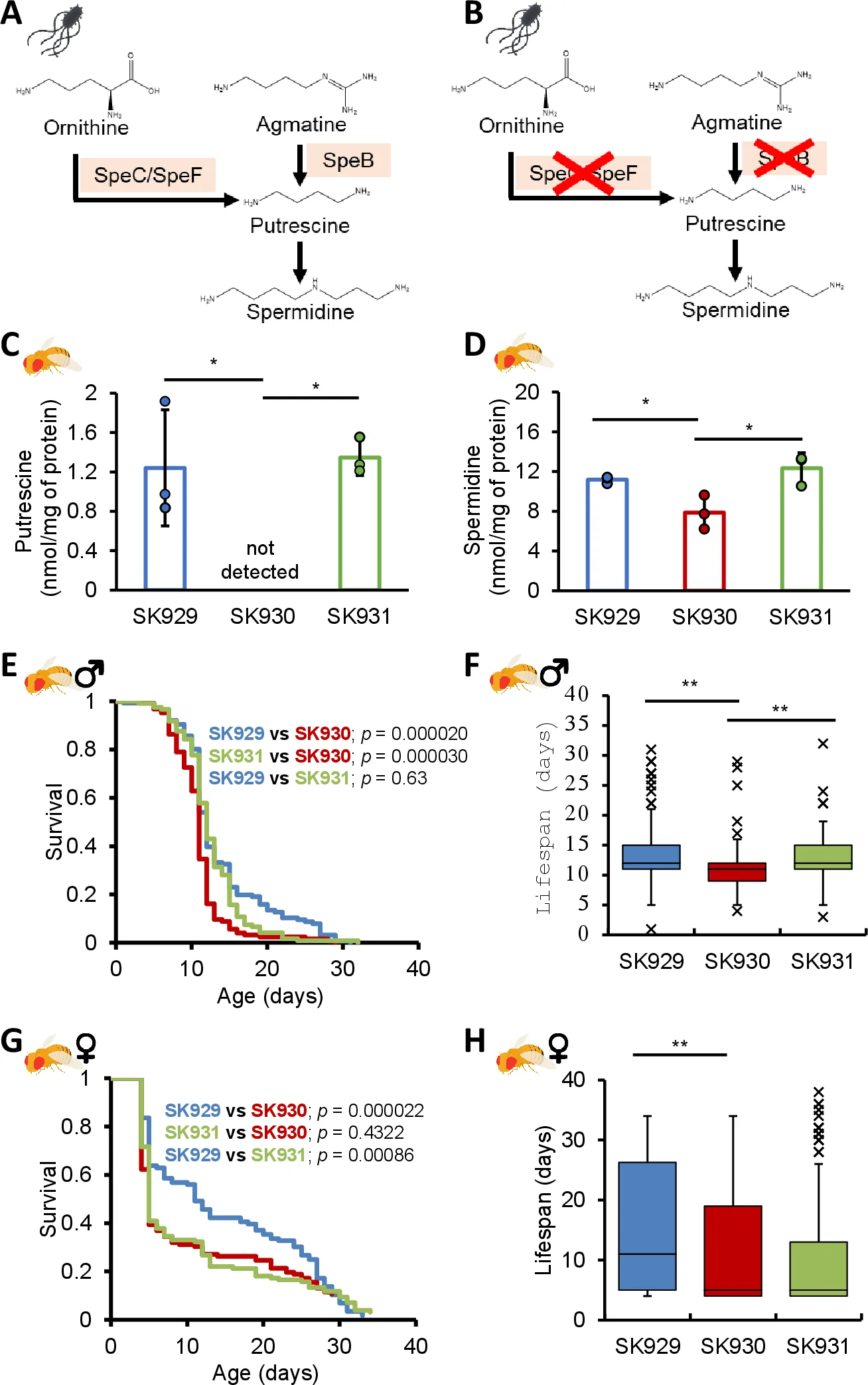
Lifespan extension by polyamines derived from gut bacteria. (**A**) Polyamine biosynthetic pathway in wild-type *E. coli.* The *speB* (agmatine ureohydrolase) pathway degrades agmatine to produce urea and putrescine; the *speC* (constitutive ornithine decarboxylase) and *speF* (inducible ornithine decarboxylase) pathways decarboxylate ornithine to produce putrescine. (**B**) Polyamine-deficient *E. coli* SK930. In polyamine-deficient *E. coli* SK930, the genes encoding all enzymes involved in the biosynthesis of putrescine (*speB*, *speC*, and *speF*) were deleted. In complemented strain SK931, a single-copy plasmid expressing *speB*, *speC*, and *speF* from native promoters was introduced into polyamine-deficient *E. coli* SK930. (**C**) Putrescine concentration in the whole body of flies monocolonized with SK929 (pBelobac11/MG1655; wild-type *E. coli* capable of producing putrescine), SK930 (pBelobac11/MG1655 except Δ*speB*, Δ*speC*, and Δ*speF*; *E. coli* mutant incapable of producing putrescine), or SK931 (pBelobac11-*speF*^+^*potE*^+^-*ygqB*^+^*speA*^+^*B*^+^-*speC*^+^/MG1655 Δ*speB*, Δ*speC*, and Δ*speF; E. coli* complemented strain capable of producing putrescine) was measured using high-performance liquid chromatography (HPLC) and standardized to protein levels. The blue and green bars show putrescine concentration in flies monocolonized with either SK929 or SK931, respectively. All data points are shown as dot plots (*n* = 3). (**D**) Spermidine concentration in the whole body of flies monocolonized with SK929, SK930, or SK931 was measured using HPLC and standardized to protein levels. The blue, red, and green bars show spermidine concentration in flies monocolonized with SK929, SK930, or SK931, respectively. All data points are shown as dot plots (*n* = 3).*, *P* < 0.05 (**E**) Survival curves of the groups of male flies monocolonized with SK929 (six containers were utilized for the rearing of 22, 23, 20, 19, 21, and 21 flies, respectively, yielding a total of 126 flies), SK930 (six containers were utilized for the rearing of 19, 22, 22, 20, 22, and 19 flies, respectively, yielding a total of 124 flies), or SK931 (six containers were utilized for the rearing of 21, 19, 20, 21, 20, and 20 flies, respectively, yielding a total of 121 flies) grown on a chemically defined modified polyamine-free holidic diet are shown in blue, red, and green, respectively. Differences in the shapes of the survival curves were analyzed using the generalized Wilcoxon test. (**F**) The lifespan of each group is shown as a boxplot. Whiskers are minimum and maximum values within the 1.5× interquartile range (IQR), and the crosses are extreme outliers outside the 1.5× IQR. The horizontal line within each box indicates the median life expectancy. Differences in median life expectancy were tested using the Mann–Whitney *U* test, and differences in average lifespan were tested using the Steel–Dwass test. The blue, red, and green bars show the average lifespan of the groups of male flies monocolonized with SK929, SK930, or SK931, respectively. **, *P* < 0.01. (**G**) survival curves of the groups of female flies monocolonized with SK929 (six containers were utilized for the rearing of 20, 20, 17, 22, 19, and 18 flies, respectively, yielding a total of 116 flies), SK930 (six containers were utilized for the rearing of 20, 20, 20, 21, 20, and 21 flies, respectively, yielding a total of 122 flies), or SK931 (six containers were utilized for the rearing of 20, 21, 24, 21, 20, and 21 flies, respectively, yielding a total of 127 flies) grown on a chemically defined modified polyamine-free holidic diet are shown in blue, red, and green, respectively. Differences in the shapes of the survival curves were analyzed using the generalized Wilcoxon test. (**H**) The lifespan of each group is shown as a boxplot. Whiskers are minimum and maximum values within the 1.5× IQR, and the crosses are extreme outliers outside the 1.5× IQR. The horizontal line within each box indicates the median life expectancy. Differences in median life expectancy were tested using the Mann–Whitney *U* test, and differences in average lifespan were tested using the Steel–Dwass test. The blue, red, and green bars show the average lifespan of the groups of female flies monocolonized with SK929, SK930, or SK931, respectively. **, *P* < 0.01.

To analyze the effects of polyamines derived only from gut bacteria on the lifespan of flies, we fed gnotobiotic flies monocolonized with genetically engineered *E. coli* a modified polyamine-free holidic diet based on the standard holidic diet (Table S2), but lacking propionic acid and methylparaben and adjusted to pH 7 to allow survival of *E. coli* (Table S5 and Fig. S2). *E. coli* can produce putrescine from ornithine via two ornithine decarboxylases: constitutive ornithine decarboxylase (SpeC) (25) and inducible ornithine decarboxylase (SpeF) (26). Additionally, it can also produce putrescine from agmatine via agmatine ureohydrolase (SpeB) (27) (Fig. 2A). The modified holidic diet was fed to gnotobiotic flies monocolonized with SK929 (pBelobac11/MG1655; wild-type *E. coli* capable of producing putrescine; Fig. 2A), SK930 (pBelobac11/MG1655 except Δ*speB* Δ*speC* Δ*speF*; *E. coli* mutant incapable of producing putrescine; Fig. 2B), or SK931 (pBelobac11-*speF*^+^*potE*^+^-*ygqB*^+^*speA*^+^*B*^+^-*speC*^+^/MG1655 Δ*speB* Δ*speC* Δ*speF*; complemented *E. coli* strain capable of producing putrescine). Because the purpose of this experiment was to evaluate the effects of polyamines biosynthesized by gut bacteria rather than the effects of bacterial colonization itself, flies monocolonized with the polyamine-deficient strain SK930 were used as the control group for flies monocolonized with the polyamine-producing strains either SK929 or SK931. In order to verify whether *E. coli* had become colonized in the gnotobiotic flies, SK929, SK930, or SK931 were mono-colonized in the germ-free flies. The flies were then fed a modified holidic diet for 24 or 72 h. Following this, the homogenates of the flies that had been mono-colonized with *E. coli* strains were serially diluted and then spread on LB agar plates containing 15 μg/mL chloramphenicol. The number of colonies was used to calculate the number of viable bacteria per fly (colony-forming unit [CFU]/fly). After a 24 h period of colonization, the number of viable *E. coli* was approximately 10^7^ (CFU/fly) in the mono-colonized gnotobiote flies, and after 72 h, the level decreased to approximately 10^6^ (CFU/fly; Fig. S3). Accordingly, *E. coli* strains were administered every 72 h with the objective of achieving permanent colonization of *E. coli* in the fly’s intestinal tract.

**Fig 3:**
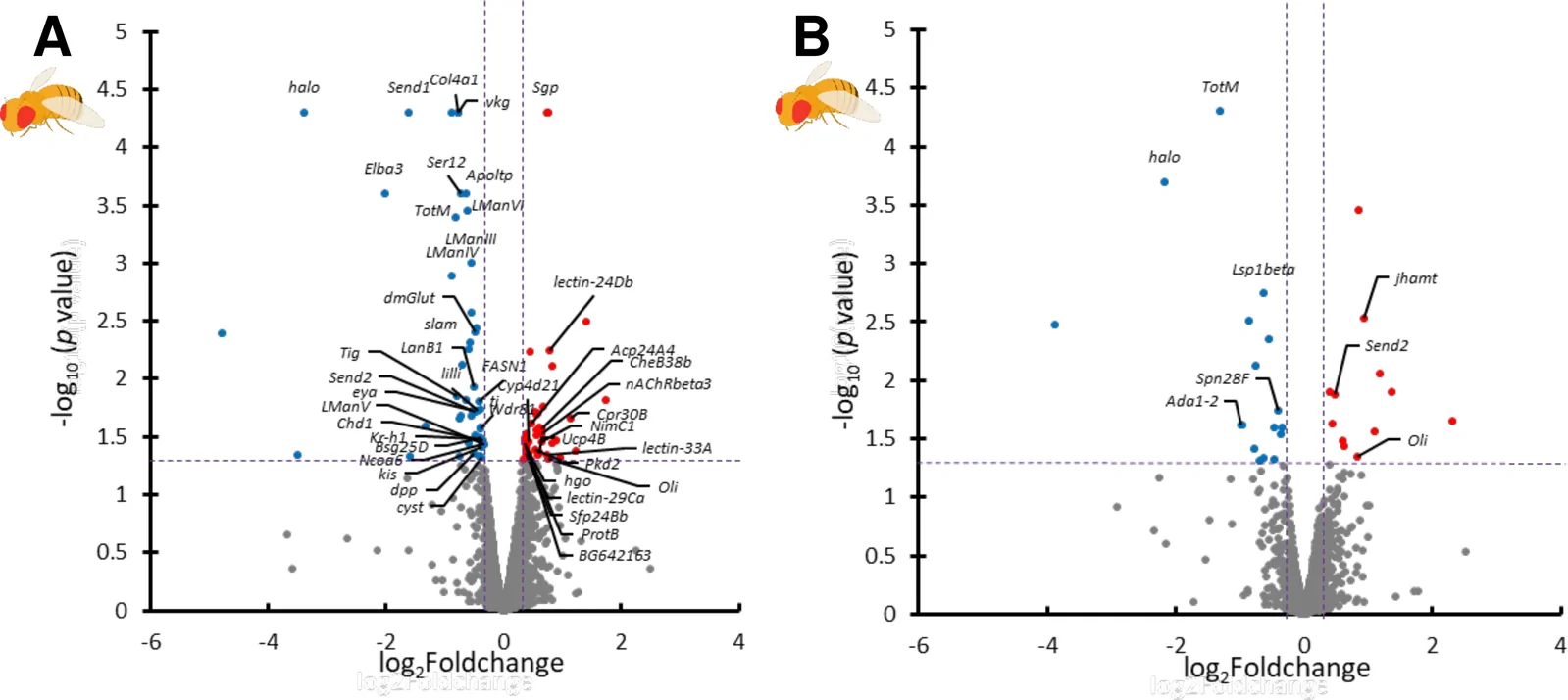
Effect of polyamines derived from gut bacteria on gene expression in the host. (**A**) Volcano plots showing differentially expressed mRNAs between the group of flies monocolonized with *E. coli* SK929 (polyamine producer: wild-type strain) and that monocolonized with SK930 (polyamine-deficient strain). Fold-change was calculated by dividing the number of mRNAs in flies monocolonized with SK929 by that in flies monocolonized with SK930, and *P*-values were determined using Cuffdiff from three different experiments. The horizontal dashed line shows −log_10_ (*P* value) = 1.30 (*P* = 0.05), and the vertical dashed lines show log_2_ (fold change) = −0.3 and + 0.3. Red and blue dots indicate genes that were up- or downregulated in flies monocolonized with SK929 compared to those with SK930. (**B**) Volcano plots showing differentially expressed mRNAs between the group of flies monocolonized with SK930 (polyamine-deficient strain) and that monocolonized with SK931 (polyamine producer: complemented strain of SK930). Fold change was calculated by dividing the number of mRNAs in flies monocolonized with SK931 by that in flies monocolonized with SK930, and *P*-values were determined using Cuffdiff from three different experiments. The horizontal dashed line shows −log_10_ (*P* value) = 1.3 (*P* = 0.05), and the vertical dashed lines show log_2_ (fold change) = −0.3 and + 0.3. Red and blue dots indicate genes that were up- or down-regulated in flies monocolonized with SK931 compared to those with SK930.

### Lifespan-extending effects of exogenous polyamines exclusively derived from gut bacteria

The concentration of putrescine in the homogenates of flies monocolonized with either SK929 or SK931, which can produce putrescine, was approximately 1.2 nmol/mg or 1.3 nmol/mg, respectively, but it was below the detection limit in the homogenates of flies monocolonized with SK930, which cannot produce polyamines (Fig. 2C). This suggests that putrescine derived from gut bacteria was absorbed by the host, and the concentration of putrescine increased in the host body. The concentrations of spermidine in the homogenates of flies monocolonized with SK929, SK930, or SK931 were approximately 11.1, 7.8, and 12.3 nmol/mg, respectively (Fig. 2D). Because flies themselves can synthesize polyamines, a basal level of spermidine was detected even in flies monocolonized with the polyamine-deficient strain SK930. In contrast, putrescine was below the detection limit in SK930-monocolonized flies, likely because the intracellular putrescine pool is small in flies. These results suggest that putrescine produced by gut bacteria was absorbed into the body of gnotobiotic flies and converted to spermidine. Notably, spermine was not detected in any group of gnotobiotic flies (data not shown).

The shapes of the survival curves of the three groups of gnotobiotic flies were compared to analyze the effects of polyamines derived from gut bacteria on the host lifespan. The survival curves were significantly different between the groups of male flies monocolonized with either SK929 or SK931, both of which produce polyamines, and those monocolonized with SK930, which cannot produce polyamines (*P =* 2.0 × 10^−5^ and *P =* 3.0 × 10^−5^, respectively; Fig. 2E). However, no significant difference in the shapes of the survival curves (*P =* 0.63, Fig. 2E) was observed between the groups of male flies monocolonized with either SK929 or SK931, both of which produce polyamines. The average lifespan of the group of male flies monocolonized with SK929 (13.8 days) or SK931 (12.6 days) was significantly longer than that of flies monocolonized with SK930 (11.0 days), which cannot produce polyamines (*P =* 6.8 × 10^−5^ and *P =* 0.00010, respectively; Fig. 2F). There was no significant difference in the average lifespan (*P =* 0.88) of the groups of male flies monocolonized with either SK929 or SK931. Furthermore, the median life expectancy was 12 days in both the SK929- and SK931-monocolonized male groups and was significantly higher than that in the SK930-monocolonized group (11 days; *P* = 2.3 × 10^−5^ and *P* = 3.4 × 10^−5^, respectively; Fig. 2F), according to the Mann–Whitney *U* test. No significant difference in the median life expectancy (*P* = 0.63; Fig. 2F) was observed between the groups of male flies monocolonized with either SK929 or SK931, both of which can produce putrescine. In female flies, the survival curves were significantly different between the groups monocolonized with SK929 and SK930 (*P* = 2.2 × 10^−5^; Fig. 2G). In contrast, no significant difference was observed between the groups monocolonized with SK931 and SK930 (*P* = 0.43; Fig. 2G). The SK929 and SK931 groups also differed (*P* = 0.00086; Fig. 2G). The average lifespan of the group of female flies monocolonized with SK929 (14.9 days) was significantly longer than that of the group monocolonized with SK930 (11.0 days, *P* = 0.00069; Fig. 2H). However, no significant difference was observed between the groups of female flies monocolonized with SK930 (11.0 days) and SK931 (10.7 days; *P* = 0.70; Fig. 2H). Furthermore, the median life expectancy of the groups of female flies monocolonized with SK929 (11 days), SK930 (5 days), or SK931 (5 days; Fig. 2H) was significantly higher in the SK929-monocolonized group than in the SK930-monocolonized group (*P* = 0.00024, Mann–Whitney *U* test), whereas no significant difference was observed between the SK930- and SK931-monocolonized groups (Fig. 2H).

### Effects of polyamines from gut bacteria on the host transcriptome

The gene expression levels in the whole body of flies monocolonized with SK929, SK930, or SK931 were analyzed by RNA sequencing (RNA-seq). The expression level of 0.41% of genes was significantly different between the group of flies monocolonized with SK929, a polyamine producer, and that monocolonized with SK930, a polyamine-deficient strain (Fig. 3A). Similarly, the expression level of 0.13% of genes was significantly different between the group of flies monocolonized with SK931, also a polyamine producer, and that monocolonized with SK930 (Fig. 3B). The expression of *TotM* and *Halo* genes was decreased in the groups of flies monocolonized with either SK929 or SK931 compared with that of the group monocolonized with SK930 (Fig. 3A and B). Furthermore, the expression of gene *CG43725* was most downregulated in the groups of flies monocolonized with either SK929 or SK931, polyamine producers, compared with that of flies monocolonized with SK930, a polyamine-deficient strain. However, the exact function of CG43725 remains unclear.

## DISCUSSION

In recent years, studies have been conducted to analyze the effects of specific gut bacterial metabolites on the host by monocolonizing germ-free animals with genetically engineered gut bacteria. For example, it has been reported that tyramine synthase-deficient strains of *Providencia alcalifaciens-*colonized nematodes showed weakened repellent behavior to octanol (28). In addition, a study on gnotobiotic mice colonized with genetically engineered *Clostridium sporogenes* revealed that aromatic amino acids and short-chain fatty acids produced by gut bacteria were involved in the host intestinal barrier function and immune response (29). However, there have been no reports on the colonization of genetically engineered gut bacteria, whose production of specific metabolites was controlled by genetic manipulation, and evaluation of the effects of the presence or absence of gut bacteria metabolites on the lifespan of the host. The present study is the first, to the best of our knowledge, to clearly show that polyamines, metabolites of gut bacteria, contribute to the lifespan extension of the host. *E. coli* produces a variety of metabolic products, and in this study, the lifespan of flies that had been monocolonized with SK930 (Δ*speAB* Δ*speC* Δ*speF* harboring an empty vector pBelobac11), where all the genes encoding *E. coli* enzymes for polyamine production had been deleted, was compared to the lifespan of flies that had been monocolonized with SK929 (wild-type strain harboring an empty vector) and SK931 (Δ*speAB* Δ*speC* Δ*speF* harboring pBelobac11-*speA*^+^*B*^+^-*speC*^+^-*speF*^+^), which are able to produce polyamines. This methodology, which differs from the method of comparing germ-free animals with animals that have been monocolonized with specific bacteria, makes it possible to analyze the impact of specific metabolites (in this study, polyamines) on lifespan.

It has been established that exogenous polyamines are derived from dietary sources and intestinal bacteria; however, the extent of their biological effects has not been fully compared. In the first half of this study, the lifespan extension by polyamines derived exclusively from the diet was analyzed by comparing the lifespan of germ-free flies fed polyamine-containing diets to those fed polyamine-free diets. In the second half of the study, the lifespan extension by polyamines derived exclusively from intestinal bacteria was analyzed by comparing the lifespan of flies monocolonized with *E. coli* strains that could (SK929 and SK931) to those that could not (SK930). The results of this study indicated that the lifespan extension effect of the presence or absence of polyamines was approximately 10% in both experiments, suggesting that the lifespan extension effect of polyamines derived from the diet and intestinal bacteria is almost the same. In a previous study, the lifespan extension effect was analyzed by feeding 5 mM spermidine to conventional flies (22). However, in this study, 10 mM spermidine was fed to germ-free flies, considering that polyamines are not produced by intestinal bacteria. The minimum amount of polyamine required to achieve a lifespan extension by polyamines derived exclusively from the diet is an issue for future research.

In this study, the genes whose expression was downregulated in flies (flies monocolonized with either SK929 or SK931) provided with polyamines by gut bacteria were *TotM* and *Halo. TotM* is involved in the JAK/STAT pathway (30). In the gut of *Drosophila* species, JAK-STAT pathway activity increases with age (31), leading to increased cytokine production, which in turn leads to a reduced lifespan. Moreover, *TotM* is an immune/stress response gene, and it has been previously described that *TotM*-knockdown flies had increased survival compared with that of control flies (32). Therefore, it is possible that downregulation of *TotM* in flies monocolonized with polyamine-producing *E. coli* might have resulted in a prolonged lifespan by modulating the immune response. *Halo* is known to be expressed during embryogenesis and is involved in the transport of lipid droplets (33). However, all flies used in the present study were adults; therefore, the reason for the reduced expression of the *Halo* gene in flies monocolonized with polyamine-producing *E. coli* strain is unknown. Conducting future research on the knockout or knockdown of fly genes will facilitate the acquisition of detailed knowledge regarding the mechanism of the lifespan-extending effect of polyamines derived from intestinal bacteria. Genetic validation of these candidate genes using knockout or RNAi fly lines will be an important subject for future study.

## MATERIALS AND METHODS

### Bacterial strains used in the present study

The list of strains used in the present study is provided in Table S1. SK929 was constructed by cloning the vacant vector pBelobac11 into wild-type *E. coli* (MG1655). SK922 is a triple-deficient strain lacking all genes encoding putrescine biosynthetic enzymes, agmatine ureohydrolase (*speB*), and ornithine decarboxylase (*speC* and *speF*) (34). SK930 was constructed by introducing pBelobac11 into SK922.

pBelobac11-*speF*^+^ was constructed as follows: a 2,499-base-pair (bp) DNA fragment including the open reading frame (ORF) of *speF* and 300-bp upstream region of *speF* of *E. coli* MG1655 was amplified by polymerase chain reaction (PCR) using KOD plus polymerase (Toyobo; product no. KOD-201), “clone_speF_FWD_BamHI” and “clone_speF_REV_BamHI” as primers, and the chromosomal DNA of MG1655 as template. The amplified 2,499-bp fragment was introduced into pBelobac11 cleaved by *Bam*HI using the In-Fusion HD Cloning Kit (Clontech-Takara; product no. 639650). The resulting plasmid was designated pBelobac11-*speF*^+^. The cloned region was sequenced, and no mutations were detected.

pBelobac11-*speF*^+^-*ygqB*^+^*speA*^+^*B*^+^ was constructed as follows: a 3,475-bp DNA fragment including the ORF of *speAB* and 300-bp upstream region including the *yqgB* gene of *E. coli* MG1655 was amplified by PCR using KOD plus polymerase, “clone_ yqgB_FWD_PciI” and “clone_speAB_REV_PciI” as primers, and the chromosomal DNA of MG1655 as template. The amplified 3,475-bp fragment was introduced into pBelobac11-*speF*^+^ cleaved by *Pci*I using the In-Fusion HD Cloning Kit. The resulting plasmid was designated pBelobac11-*speF*^+^-*ygqB*^+^*speA*^+^*B*^+^. The cloned region was sequenced, and no mutations were detected.

pBelobac11-*speF*^+^-*ygqB*^+^*speA*^+^*B*^+^-*speC*^+^ was constructed as follows: a 2,436-bp DNA fragment including the ORF of *speC* and 300-bp upstream region of *speC* of *E. coli* MG1655 was amplified by PCR using KOD plus polymerase, “clone_speC_FWD_XhoI” and “clone_speC_REV_XhoI” as primers, and the genomic DNA of MG1655 as template. The amplified 2,436-bp fragment was introduced into pBelobac11-*speF*^+^-*ygqB*^+^*speA*^+^*B*^+^ cleaved by *Xho*I using the In-Fusion HD Cloning Kit. The resulting plasmid was designated as pBelobac11-*speF*^+^*-ygqB*^+^*speA*^+^*B*^+^-*speC*^+^. The cloned region was sequenced, and no mutations were detected.

pBelobac11-*speF*^+^*potE*^+^*-ygqB*^+^*speA*^+^*B*^+^-*speC*^+^ was constructed as follows: a 3,815-bp DNA fragment including the ORF of *speFpotE* and 300-bp upstream region of *speFpotE* of *E. coli* MG1655 was amplified by PCR using KOD plus polymerase, “clon_speFpotE_F_NotI” and “clon_speFpotE_R_NotI” as primers, and the chromosomal DNA of MG1655 as template. The amplified 3,815-bp fragment was introduced into pBelobac11-*speF*^+^*-ygqB*^+^*speA*^+^*B*^+^-*speC*^+^ cleaved by *Not*I using the In-Fusion HD Cloning Kit to eliminate *speF* of this plasmid. The resulting plasmid was designated pBelobac11-*speF*^+^*potE*^+^*-ygqB*^+^*speA*^+^*B*^+^-*speC*^+^. The cloned region was sequenced, and no mutations were detected.

SK931 cells were constructed by introducing pBelobac11-*speF*^+^*potE*^+^*-ygqB*^+^*speA*^+^*B*^+^-*speC*^+^ into SK922.

### Culture of *E. coli*

Chloramphenicol was added at a final concentration of 15 µg/mL to 10 mL of LB broth in a 50 mL sterile conical tube with a lid (Greiner; product no. 227261). SK929 (wild-type strain), SK930 (polyamine-deficient strain), or SK931 (complemented strain of SK930) was inoculated from the glycerol stock using a sterile toothpick and incubated at 37°C (Advantech; product no. TVA460DA) at 140 rpm on a reciprocal shaker (TAITEC; product no. NR-1) overnight.

### Generation of gnotobiotic flies and determination of viable bacteria in their bodies

Oregon-R flies were used in all experiments. Germ-free flies were generated by embryo surface sterilization using a sodium hypochlorite-based disinfectant, based on the general procedure described previously (35). Flies were maintained under sterile conditions throughout development. Germ-free status was assessed by CFU assays of representative fly homogenates as described previously (35). Briefly, representative flies were homogenized under aseptic conditions, plated on culture medium, and incubated to confirm the absence of detectable bacterial growth. Gnotobiotic flies were generated from these germ-free flies and thereafter handled and maintained under aseptic conditions. Flies that had been colonized with *E. coli* were anesthetized with diethyl ether, and the bacteria on the fly surface were killed by immersion in 1 mL of 70% ethanol on a sterile dissecting dish. The 70% ethanol was then removed using Kimwipes paper, and the remaining ethanol was washed away with 1 mL of sterile PBS. The sterile PBS was then removed using Kimwipes paper. Subsequent to this, the 30 washed gnotobiotic flies were placed in a 2 mL screw-cap tube with microbeads and 500 μL of sterile PBS and homogenized using a 3D homogenizer (Bertin Technologies; product no. PR240-A) at 4,000 rpm for 2 min. A 100-µL aliquot of the homogenate, serially diluted in sterile PBS, was spread onto LB plates containing 15 µg/mL chloramphenicol and incubated at 37°C overnight. The number of colonies that appeared on the LB plates was used to calculate the number of viable bacteria (CFU/fly) in the bodies of the gnotobiotic flies.

### Diet preparation and administration

Our standard fly diet (40 g/L corn flour, 5.5 g/L lyophilized agar, 100 g/L glucose, 80 g/L dried beer yeast, 720 mg/L butyl benzoate [butyl benzoate was dissolved in ethanol at a concentration of 15% {wt/vol} and added to the diet at a final concentration of 720 mg/L], and 3.8 mL/L propionic acid) contained polyamines (Fig. S1); therefore, it was not suitable for comparative analysis of health-promoting effects of polyamines derived from diet and gut bacteria. For this reason, we used a chemically defined holidic diet, which was prepared as previously reported and is polyamine-free (24), and supplemented with spermidine (10 mM, final concentration) or putrescine (15 mM, final concentration). Approximately 5.5 mL of the diet was dispensed in polystyrene *Drosophila* vials (Genesee Scientific; product no. 32-116) capped with *Drosophila* Vial Caps (Genesee Scientific; product no. 49-102). Sterilization was carried out by autoclaving or gamma-ray irradiation using Koga Isotope (http://www.koga-isotope.co.jp/; Kōka City, Japan). *E. coli* is unable to grow in the normal holidic diet because of the presence of preservatives and low pH (Fig. S2A and B). Therefore, to maintain *E. coli* in the gastrointestinal tract of the flies, a modified holidic diet with pH adjusted to 7 using sodium hydroxide (NaOH) and non-supplemented with preservatives (propanoic acid and methylparaben) was fed to gnotobiotic flies monocolonized with *E. coli*.

### Preparation of gnotobiotic flies

Gnotobiotic flies were generated from the germ-free flies described above. The cultures of *E. coli* strains grown in modified holidic broth supplemented with 15 µg/mL chloramphenicol were centrifuged at 1,449 × g for 15 min at 25°C. The supernatant was removed by decantation, and the cells were resuspended in 5 mL of filter-sterilized phosphate-buffered saline (PBS) supplemented with 200 µg/mL thiamine (Fujifilm Wako Pure Chemicals; product no. 201-00852). The optical density at 600 nm (OD_600_) of the suspension was adjusted to 10 using PBS supplemented with 200 µg/mL thiamine. Fifty microliters of the bacterial suspension was added to the surface of the modified holidic diet, and the surface was allowed to dry on a clean bench for 30 min. Chloramphenicol was included to maintain the plasmids harbored by the *E. coli* strains and was applied equally to all three groups. Within 24 h after hatching, approximately 30 sterile *D. melanogaster* flies per vial were transferred to a diet containing *E. coli*. The flies were monocolonized with each *E. coli* strain through oral intake via diet ingestion. Three vials were prepared for each *E. coli* strain. Rearing was carried out in an incubator at 29°C in the dark and 40%–60% humidity.

### Analysis of *D. melanogaster* lifespan

For the dietary polyamine experiment, germ-free flies were fed holidic diets supplemented with putrescine, supplemented with spermidine, or without polyamines. For the gnotobiotic lifespan experiment, flies were monocolonized with SK929 (wild-type strain), SK930 (polyamine-deficient strain), or SK931 (complemented strain of SK930) and fed the modified holidic diet. In all lifespan experiments, the fly diet was replaced every 3 or 4 days, and the flies were not anesthetized during the entire experimental period. Flies were maintained in vials during the lifespan experiments. In the gnotobiotic lifespan experiment, male and female flies were analyzed separately.

### Polyamines quantification by high-performance liquid chromatography

To quantify polyamines in *D. melanogaster*, the flies were anesthetized with carbon dioxide and washed with 70% ethanol and PBS. Thirty flies were washed, and approximately 100 µL of microbeads (Tohshin Riko; product no. 080) and 300 µL of PBS were added to a 2 mL screw-cap tube. The flies were disrupted using a three-dimensional homogenizer (Bertin Technologies; product no. PR240-A) at 6,000 rpm for 2 min and centrifuged at 18,717 × *g* for 10 min at 4°C. The supernatant (200 μL) was mixed with 20 μL of 100% trichloroacetic acid (Nacalai Tesque; product no. 34637-85) and centrifuged at 18,717 × *g* for 10 min at 4°C. The supernatant obtained from the previous centrifugation step was filtered using Cosmonice Filter W (Nacalai Tesque; product no. 06543-04) before high-performance liquid chromatography (HPLC) analysis. Polyamines were separated using a cation-exchange column (Hitachi; product no. #2619PH, 4.6 × 50 mm) in normal-phase mode; HPLC was performed as previously described (36). Protein debris resulting from centrifugation was dissolved in 0.1 N NaOH, and the protein concentration was determined by the Bradford method using a Bio-Rad protein assay kit (Bio-Rad Laboratories; product no. #5000006JA). The protein-standardized polyamine concentration (nmol/mg of protein) was determined by dividing the polyamine concentration determined using HPLC by the protein concentration determined using the Bradford method.

### RNA sequencing

The total RNA used for RNA-seq was obtained from samples 14 days after single fixation of SK929 (wild-type strain), SK930 (polyamine-deficient strain), or SK931 (complemented strain of SK930). Three groups, each containing 10 flies, were analyzed for each strain. Total RNA was prepared as per the manufacturer’s protocol using Sepasol-RNA I Super G (Nacalai Tesque, product no. 09379-55). Library preparation was performed using the TruSeq Stranded mRNA kit (Illumina Inc., CA, USA). Pair-end RNA-seq was performed in triplicate for each condition on the HiSeq 2500 platform (Illumina Inc.) at Macrogen (Kyoto, Japan) to obtain the sequencing data. Paired-end read sequences were quality checked using FASTQC (37) and mapped to the genome sequence of *D. melanogaster*, obtained from Ensembl release 107 (38) using HISAT2 version 2.2.1 (39). The resulting BAM files were sorted using SAMtools version 1.15 (40). Finally, the expression levels and significant differences for each gene were calculated using Cuffdiff version 2.1.1 (41).

### Statistical analyses

The Kaplan–Meier method was used to generate the survival curves of different groups fed holidic diets supplemented with polyamines and monocolonized with *E. coli* strains. Differences in the shapes of the obtained survival curves were analyzed using the generalized Wilcoxon test. Because lifespan was evaluated as day-based survival data and was not assumed to follow a normal distribution, non-parametric tests were used throughout this study.

For pairwise comparisons of median lifespan between two groups, the Mann–Whitney *U* test was used. Differences in the average lifespan were tested using the Steel–Dwass method. Excel statistics (Social Information Service Co., Ltd., Japan) were used for all statistical analyses.

## Data Availability

The RNA-seq data were registered in DDBJ as DRA021858.

## SUPPLEMENTAL MATERIAL

Supplemental material mbio.01484-26-s0001.pdf Supplemental figures and tables.

ASM does not own the copyrights to Supplemental Material that may be linked to, or accessed through, an article. The authors have granted ASM a non-exclusive, world-wide license to publish the Supplemental Material files. Please contact the corresponding author directly for reuse.
