# Supplementary material for: Extension of host lifespan by polyamines derived from gut bacteria
Source: mBio. 2026 Sep 10;17(10):e01484-26. doi: 10.1128/mbio.01484-26 (PMC13712837; doi:10.1128/mbio.01484-26)

Extended Data Figure 1 Comparison of polyamine concentration of existing diet and Holidic diet

The concentration of polyamines in the existing fly diet (Normal diet, germ-free fly diet (1), germ-free fly diet (2)) and Holidic diet was measured using HPLC. Putrescine, spermidine, and spermine are shown in orange, blue, and green, respectively. All data points are shown as dot plots (n = 3).

A, it was impossible to determine the putrescine concentration because another peak overlapped the putrescine peak on the chromatogram in our HPLC system.

Extended Data Figure 2 Examination of the growth of *E. coli* on the Holidic diet

A. *E. coli* were grown in LB medium supplemented with 15 µg/mL chloramphenicol and preservatives (0.6% (w/w) propanoic acid, 1.5% (w/w) methylparaben, or simultaneous supplementation of 0.6% (w/w) propanoic acid and 1.5% (w/w) methylparaben, all contained in the Holidic diet), with reciprocal shaking at 140 rpm overnight at 37 °C and growth was measured. The OD_{600} values of SK929 (polyamine producer: wild-type strain), SK930 (polyamine deficient strain), or SK931 (polyamine producer: the complemented strain of SK930) are shown in blue, red, and green, respectively.

B. *E. coli* were grown without preservatives in a Holidic broth, with pH adjusted to 4, 5, 6, or 7, with reciprocal shaking at 140 rpm overnight at 37 °C, and growth was measured. The OD_{600} values of SK929, SK930, or SK931 are shown in blue, red, and green, respectively.

Extended Data Figure 3 Determination of viable bacteria in bodies of flies

The bacteria on the body surface of gnotobiotic flies that had been mono-colonized with *E. coli* strains were sterilized with 70% ethanol, then the ethanol was removed and the flies were homogenized using a 3D homogenizer. The resulting homogenate was serially diluted and spread onto LB agar plates containing chloramphenicol. The number of viable chloramphenicol-resistant bacteria inside the flies was determined by colony counting and is shown in the bar graph. The preparation of fly homogenate was carried out at 24 and 72 hours after the mono-colonization of SK929 shown in blue (polyamine producer: wild-type strain), SK930 shown in red (polyamine deficient strain), or SK931 shown in green (polyamine producer: the complemented strain of SK930).

Extended Data Table 1. Plasmids and oligonucleotides used in this study

Strains, plasmids, and oligonucleotides	Genotype	Source or reference
**Strains**		
MG1655	*Escherichia coli* K-12 F$^-$ prototrophic	Laboratory stock
SK922	MG1655 except Δ*speB*:FRT Δ*speC*::FRT Δ*speF*::FRT	(34)
SK929	pBelobac11/MG1655	This study
SK930	pBelobac11/SK922	(34)
SK931	pBelobac11-*speF*$^+$*potE*$^+$-*ygqB*$^+$*speA*$^+$*B*$^+$-*speC*$^+$/SK922	This study
**Plasmids**		
pBelobac11	Mini-F replicon cat$^+$	New England Biolabs, MA
pBelobac11-*speF*$^+$	Mini-F replicon *cat*$^+$ *speF*$^+$	This study
pBelobac11-*speF*$^+$-*ygqB*$^+$*speA*$^+$*B*$^+$	Mini-F replicon *cat*$^+$ *speF*$^+$ *ygqB*$^+$*speA*$^+$*B*$^+$	This study
pBelobac11-*speF*$^+$-*ygqB*$^+$*speA*$^+$*B*$^+$-*speC*$^+$	Mini-F replicon *cat*$^+$ *speF*$^+$*ygqB*$^+$*speA*$^+$*B*$^+$*speC*$^+$	This study
pBelobac11-*speF*$^+$*potE*$^+$-*ygqB*$^+$*speA*$^+$*B*$^+$-*speC*$^+$	Mini-F replicon *cat*$^+$ *speF*$^+$*potE*$^+$*ygqB*$^+$*speA*$^+$*B*$^+$*speC*$^+$	This study
**Oligonucleotides (5' to 3')**		
clone_speF_FWD_BamHI	cggtacccggggatctcatcgcaatagctt	
clone_speF_REV_BamHI	cgactctagaggatctcataatttttcccctttcaac	
clone_ yqgB_FWD_PciI	gcaagtattgacatgtgaaatcagagttttggc	
clone_speAB_REV_PciI	ggttacgacgacatgttactcgcccttttt	
clone_speC_FWD_XhoI	gggcgaagccctcgaagcgaaaggtgccat	
clone_speC_REV_XhoI	tcctcgctcactcgattacttcaacacata	
clon_speFpotE_F_NotI	atattcgatgcggcctcatcgcaatagcttcgact	
clon_speFpotE_R_NotI	acggcccgggcggccttaaccgtgtttatttttca	

Extended Data Table 2. Formulation of Holidic diet, which is polyamine-free.

Ingredient	Product number and source of the solution	Final concentration	
Sucrose	NACALAI TESQUE (30403-55)	1.71	% (w/v)
L-isoleucine	Sigma (I2752)	0.18	% (w/v)
L-leucine	Sigma (L8912)	0.12	% (w/v)
L-tyrosine	Sigma (T3754)	0.042	% (w/v)
glacial acetic acid	FUJIFILM Wako Pure Chemical Corporation (017-00251)	0.3	% (v/v)

KH_2PO_4	KANTO CHEMICAL CO., INC. (3237923)	0.3	% (w/v)
$NaHCO_3$	NACALAI TESQUE (31213-15)	0.1	% (w/v)
$CaCl_2$ $2H_2O$	NACALAI TESQUE (08895-15)	0.0168	% (w/v)
$CuSO_4$ $5H_2O$	NACALAI TESQUE (09605-04)	0.00025	% (w/v)
$FeSO_4$ $7H_2O$	KANTO CHEMICAL CO., INC. (16038-30)	0.0025	% (w/v)
$MgSO_4$ (anhydrous)	NACALAI TESQUE (21032-95)	0.025	% (w/v)
$MnCl_2$ $4H_2O$	NACALAI TESQUE (21211-32)	0.0001	% (w/v)
$ZnSO_4$ $7H_2O$	Sigma (Z0251)	0.0025	% (w/v)
Cholesterol	FUJIFILM Wako Pure Chemical Corporation (034-03002)	0.01	% (w/v)
Agar powder	NACALAI TESQUE (01028-85)	2	% (w/v)
L-arginine	Sigma (A5131)	0.002562	% (w/v)
L-histidine	Sigma (H8000)	0.061	% (w/v)
L-lysine (HCl)	Sigma (L5626)	0.0549	% (w/v)
L-methionine	Sigma (M9625)	0.0488	% (w/v)
L-phenylalanine	Sigma (P2126)	0.0793	% (w/v)
L-threonine	Sigma (T8625)	0.122	% (w/v)
L-tryptophan	Sigma (T0254)	0.0305	% (w/v)
L-valine	Sigma (V0500)	0.1708	% (w/v)
L-alanine	Sigma (A7627)	0.002562	% (w/v)
L-asparagine	Sigma (A0884)	0.1037	% (w/v)
L-aspartic acid	Sigma (A6683)	0.1037	% (w/v)
L-cysteine HCl	Sigma (C1276)	0.0061	% (w/v)
L-glutamine	Sigma (G3126)	0.1525	% (w/v)
glycine	Sigma (G7126)	0.1952	% (w/v)
L-proline	Sigma (P0380)	0.0915	% (w/v)
L-serine	Sigma (S4500)	0.1159	% (w/v)
Monosodium glutamate	Sigma (G5889)	0.000255	% (w/v)
thiamine (aneurin)	FUJIFILM Wako Pure Chemical Corporation (203-00851)	0.00014	% (w/v)
riboflavin	Sigma (R4500)	0.00007	% (w/v)
nicotinic acid	Sigma (N4126)	0.00084	% (w/v)
Ca pantothenate	NACALAI TESQUE (26003-12)	0.001085	% (w/v)
pyridoxine (HCl)	Sigma (P9755)	0.000175	% (w/v)
biotin	Sigma (B4501)	0.000014	% (w/v)
Sodium folate solution	Sigma (F7876)	0.00005	% (w/v)
choline chloride	Sigma (C1879)	0.005	% (w/v)
myo-inositol	Sigma (I7508)	0.000504	% (w/v)

inosine	Sigma (I4125)	0.006504	% (w/v)
uridine	Sigma (U3750)	0.006	% (w/v)
Propionic acid	FUJIFILM Wako Pure Chemical Corporation (010-15815)	0.6	% (v/v)
4-Hydroxybenzoic acid	FUJIFILM Wako Pure Chemical Corporation (132-02635)	0.1	% (w/v)

Extended Data Table 3. Formulation of Holidic diet supplement with 15 mM putrescine

Ingredient	Product number and source of the solution	Final concentration	
Sucrose	NACALAI TESQUE (30403-55)	1.71	% (w/v)
L-isoleucine	Sigma (I2752)	0.18	% (w/v)
L-leucine	Sigma (L8912)	0.12	% (w/v)
L-tyrosine	Sigma (T3754)	0.042	% (w/v)
glacial acetic acid	FUJIFILM Wako Pure Chemical Corporation (017-00251)	0.3	% (v/v)
KH_2PO_4	KANTO CHEMICAL CO., INC. (3237923)	0.3	% (w/v)
$NaHCO_3$	NACALAI TESQUE (31213-15)	0.1	% (w/v)
$CaCl_2$ $2H_2O$	NACALAI TESQUE (08895-15)	0.0168	% (w/v)
$CuSO_4$ $5H_2O$	NACALAI TESQUE (09605-04)	0.00025	% (w/v)
$FeSO_4$ $7H_2O$	KANTO CHEMICAL CO., INC. (16038-30)	0.0025	% (w/v)
$MgSO_4$ (anhydrous)	NACALAI TESQUE (21032-95)	0.025	% (w/v)
$MnCl_2$ $4H_2O$	NACALAI TESQUE (21211-32)	0.0001	% (w/v)
$ZnSO_4$ $7H_2O$	Sigma (Z0251)	0.0025	% (w/v)
Cholesterol	FUJIFILM Wako Pure Chemical Corporation (034-03002)	0.01	% (w/v)
Agar powder	NACALAI TESQUE (01028-85)	2	% (w/v)
L-arginine	Sigma (A5131)	0.002562	% (w/v)
L-histidine	Sigma (H8000)	0.061	% (w/v)
L-lysine (HCl)	Sigma (L5626)	0.0549	% (w/v)
L-methionine	Sigma (M9625)	0.0488	% (w/v)
L-phenylalanine	Sigma (P2126)	0.0793	% (w/v)
L-threonine	Sigma (T8625)	0.122	% (w/v)
L-tryptophan	Sigma (T0254)	0.0305	% (w/v)
L-valine	Sigma (V0500)	0.1708	% (w/v)
L-alanine	Sigma (A7627)	0.002562	% (w/v)
L-asparagine	Sigma (A0884)	0.1037	% (w/v)
L-aspartic acid	Sigma (A6683)	0.1037	% (w/v)
L-cysteine HCl	Sigma (C1276)	0.0061	% (w/v)
L-glutamine	Sigma (G3126)	0.1525	% (w/v)
glycine	Sigma (G7126)	0.1952	% (w/v)

L-proline	Sigma (P0380)	0.0915	% (w/v)
L-serine	Sigma (S4500)	0.1159	% (w/v)
Monosodium glutamate	Sigma (G5889)	0.000255	% (w/v)
thiamine (aneurin)	FUJIFILM Wako Pure Chemical Corporation (203-00851)	0.00014	% (w/v)
riboflavin	Sigma (R4500)	0.00007	% (w/v)
nicotinic acid	Sigma (N4126)	0.00084	% (w/v)
Ca pantothenate	NACALAI TESQUE (26003-12)	0.001085	% (w/v)
pyridoxine (HCl)	Sigma (P9755)	0.000175	% (w/v)
biotin	Sigma (B4501)	0.000014	% (w/v)
Sodium folate solution	Sigma (F7876)	0.00005	% (w/v)
choline chloride	Sigma (C1879)	0.005	% (w/v)
myo-inositol	Sigma (I7508)	0.000504	% (w/v)
inosine	Sigma (I4125)	0.006504	% (w/v)
uridine	Sigma (U3750)	0.006	% (w/v)
Propionic acid	FUJIFILM Wako Pure Chemical Corporation (010-15815)	0.6	% (v/v)
4-Hydroxybenzoic acid	FUJIFILM Wako Pure Chemical Corporation (132-02635)	0.1	% (w/v)
Putrescine	NACALAI TESQUE (29428-14)	15	mM

Extended Data Table 4. Formulation of Holidic diet supplement with 15 mM spermidine

Ingredient	Product number and source of the solution	Final concentration	
Sucrose	NACALAI TESQUE (30403-55)	1.71	% (w/v)
L-isoleucine	Sigma (I2752)	0.18	% (w/v)
L-leucine	Sigma (L8912)	0.12	% (w/v)
L-tyrosine	Sigma (T3754)	0.042	% (w/v)
glacial acetic acid	FUJIFILM Wako Pure Chemical Corporation (017-00251)	0.3	% (v/v)
KH_2PO_4	KANTO CHEMICAL CO., INC. (3237923)	0.3	% (w/v)
$NaHCO_3$	NACALAI TESQUE (31213-15)	0.1	% (w/v)
$CaCl_2$ $2H_2O$	NACALAI TESQUE (08895-15)	0.0168	% (w/v)
$CuSO_4$ $5H_2O$	NACALAI TESQUE (09605-04)	0.00025	% (w/v)
$FeSO_4$ $7H_2O$	KANTO CHEMICAL CO., INC. (16038-30)	0.0025	% (w/v)
$MgSO_4$ (anhydrous)	NACALAI TESQUE (21032-95)	0.025	% (w/v)
$MnCl_2$ $4H_2O$	NACALAI TESQUE (21211-32)	0.0001	% (w/v)
$ZnSO_4$ $7H_2O$	Sigma (Z0251)	0.0025	% (w/v)
Cholesterol	FUJIFILM Wako Pure Chemical Corporation (034-03002)	0.01	% (w/v)
Agar powder	NACALAI TESQUE (01028-85)	2	% (w/v)
L-arginine	Sigma (A5131)	0.002562	% (w/v)

L-histidine	Sigma (H8000)	0.061	% (w/v)
L-lysine (HCl)	Sigma (L5626)	0.0549	% (w/v)
L-methionine	Sigma (M9625)	0.0488	% (w/v)
L-phenylalanine	Sigma (P2126)	0.0793	% (w/v)
L-threonine	Sigma (T8625)	0.122	% (w/v)
L-tryptophan	Sigma (T0254)	0.0305	% (w/v)
L-valine	Sigma (V0500)	0.1708	% (w/v)
L-alanine	Sigma (A7627)	0.002562	% (w/v)
L-asparagine	Sigma (A0884)	0.1037	% (w/v)
L-aspartic acid	Sigma (A6683)	0.1037	% (w/v)
L-cysteine HCl	Sigma (C1276)	0.0061	% (w/v)
L-glutamine	Sigma (G3126)	0.1525	% (w/v)
glycine	Sigma (G7126)	0.1952	% (w/v)
L-proline	Sigma (P0380)	0.0915	% (w/v)
L-serine	Sigma (S4500)	0.1159	% (w/v)
Monosodium glutamate	Sigma (G5889)	0.000255	% (w/v)
thiamine (aneurin)	FUJIFILM Wako Pure Chemical Corporation (203-00851)	0.00014	% (w/v)
riboflavin	Sigma (R4500)	0.00007	% (w/v)
nicotinic acid	Sigma (N4126)	0.00084	% (w/v)
Ca pantothenate	NACALAI TESQUE (26003-12)	0.001085	% (w/v)
pyridoxine (HCl)	Sigma (P9755)	0.000175	% (w/v)
biotin	Sigma (B4501)	0.000014	% (w/v)
Sodium folate solution	Sigma (F7876)	0.00005	% (w/v)
choline chloride	Sigma (C1879)	0.005	% (w/v)
myo-inositol	Sigma (I7508)	0.000504	% (w/v)
inosine	Sigma (I4125)	0.006504	% (w/v)
uridine	Sigma (U3750)	0.006	% (w/v)
Propionic acid	FUJIFILM Wako Pure Chemical Corporation (010-15815)	0.6	% (v/v)
4-Hydroxybenzoic acid	FUJIFILM Wako Pure Chemical Corporation (132-02635)	0.1	% (w/v)
Spermidine	NACALAI TESQUE (32110-54)	15	mM

Extended Data Table 5. Formulation of modified Holidic diet, which is polyamine-free.

Ingredient	Product number and source of the solution	Final concentration	
Sucrose	NACALAI TESQUE (30403-55)	1.71	% (w/v)
L-isoleucine	Sigma (I2752)	0.18	% (w/v)
L-leucine	Sigma (L8912)	0.12	% (w/v)

L-tyrosine	Sigma (T3754)	0.042	% (w/v)
glacial acetic acid	FUJIFILM Wako Pure Chemical Corporation (017-00251)	0.3	% (v/v)
KH_2PO_4	KANTO CHEMICAL CO., INC. (3237923)	0.3	% (w/v)
$NaHCO_3$	NACALAI TESQUE (31213-15)	0.1	% (w/v)
$CaCl_2$ $2H_2O$	NACALAI TESQUE (08895-15)	0.0168	% (w/v)
$CuSO_4$ $5H_2O$	NACALAI TESQUE (09605-04)	0.00025	% (w/v)
$FeSO_4$ $7H_2O$	KANTO CHEMICAL CO., INC. (16038-30)	0.0025	% (w/v)
$MgSO_4$ (anhydrous)	NACALAI TESQUE (21032-95)	0.025	% (w/v)
$MnCl_2$ $4H_2O$	NACALAI TESQUE (21211-32)	0.0001	% (w/v)
$ZnSO_4$ $7H_2O$	Sigma (Z0251)	0.0025	% (w/v)
Cholesterol	FUJIFILM Wako Pure Chemical Corporation (034-03002)	0.01	% (w/v)
Agar powder	NACALAI TESQUE (01028-85)	2	% (w/v)
L-arginine	Sigma (A5131)	0.002562	% (w/v)
L-histidine	Sigma (H8000)	0.061	% (w/v)
L-lysine (HCl)	Sigma (L5626)	0.0549	% (w/v)
L-methionine	Sigma (M9625)	0.0488	% (w/v)
L-phenylalanine	Sigma (P2126)	0.0793	% (w/v)
L-threonine	Sigma (T8625)	0.122	% (w/v)
L-tryptophan	Sigma (T0254)	0.0305	% (w/v)
L-valine	Sigma (V0500)	0.1708	% (w/v)
L-alanine	Sigma (A7627)	0.002562	% (w/v)
L-asparagine	Sigma (A0884)	0.1037	% (w/v)
L-aspartic acid	Sigma (A6683)	0.1037	% (w/v)
L-cysteine HCl	Sigma (C1276)	0.0061	% (w/v)
L-glutamine	Sigma (G3126)	0.1525	% (w/v)
glycine	Sigma (G7126)	0.1952	% (w/v)
L-proline	Sigma (P0380)	0.0915	% (w/v)
L-serine	Sigma (S4500)	0.1159	% (w/v)
Monosodium glutamate	Sigma (G5889)	0.000255	% (w/v)
thiamine (aneurin)	FUJIFILM Wako Pure Chemical Corporation (203-00851)	0.00014	% (w/v)
riboflavin	Sigma (R4500)	0.00007	% (w/v)
nicotinic acid	Sigma (N4126)	0.00084	% (w/v)
Ca pantothenate	NACALAI TESQUE (26003-12)	0.001085	% (w/v)
pyridoxine (HCl)	Sigma (P9755)	0.000175	% (w/v)
biotin	Sigma (B4501)	0.000014	% (w/v)
Sodium folate solution	Sigma (F7876)	0.00005	% (w/v)

choline chloride	Sigma (C1879)	0.005	% (w/v)
myo-inositol	Sigma (I7508)	0.000504	% (w/v)
inosine	Sigma (I4125)	0.006504	% (w/v)
uridine	Sigma (U3750)	0.006	% (w/v)
Glucose	FUJIFILM Wako Pure Chemical Corporation (045-31167)	5	% (w/v)
Chloramphenicol	FUJIFILM Wako Pure Chemical Corporation (036-10571)	1.5	μg/mL

The mixture was adjusted to pH 7 using NaOH.

Extended Data Figure 1

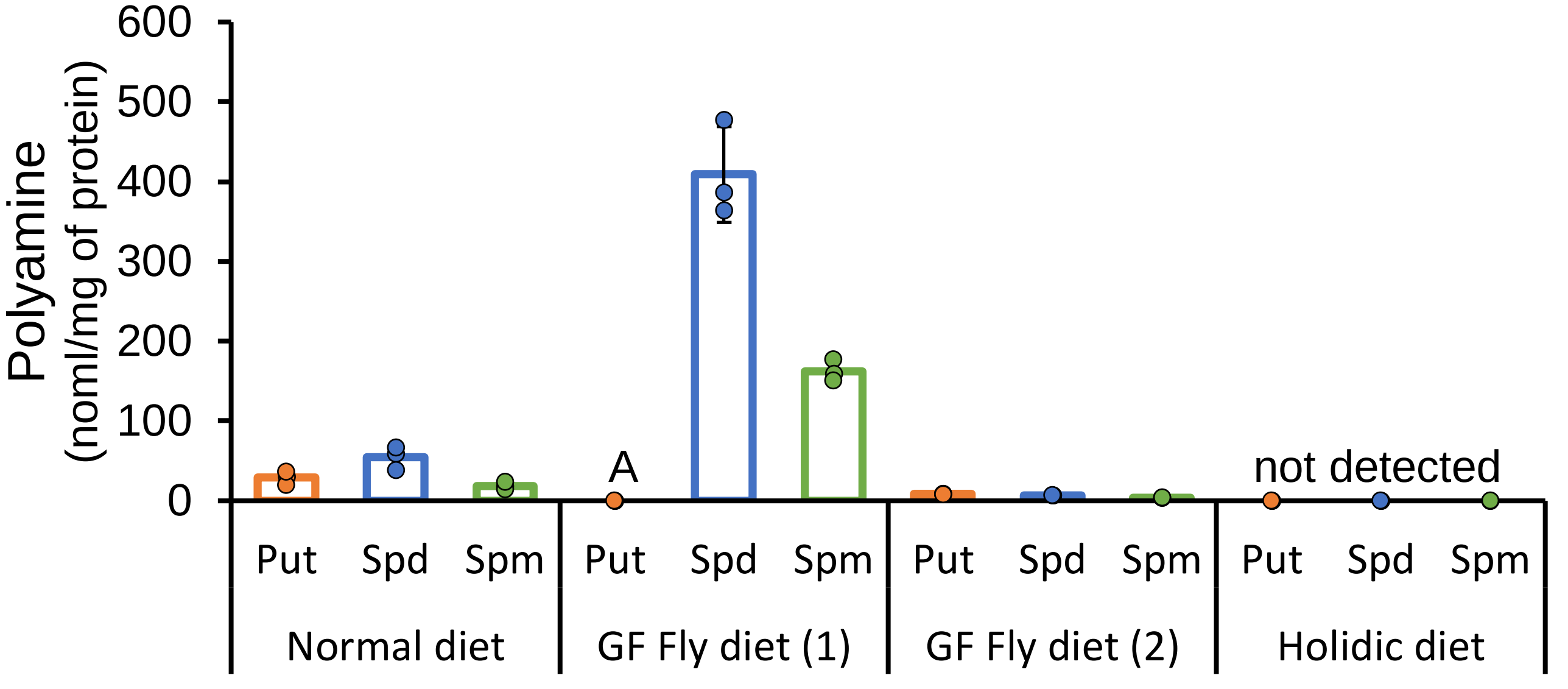

Extended Data Figure 2

A

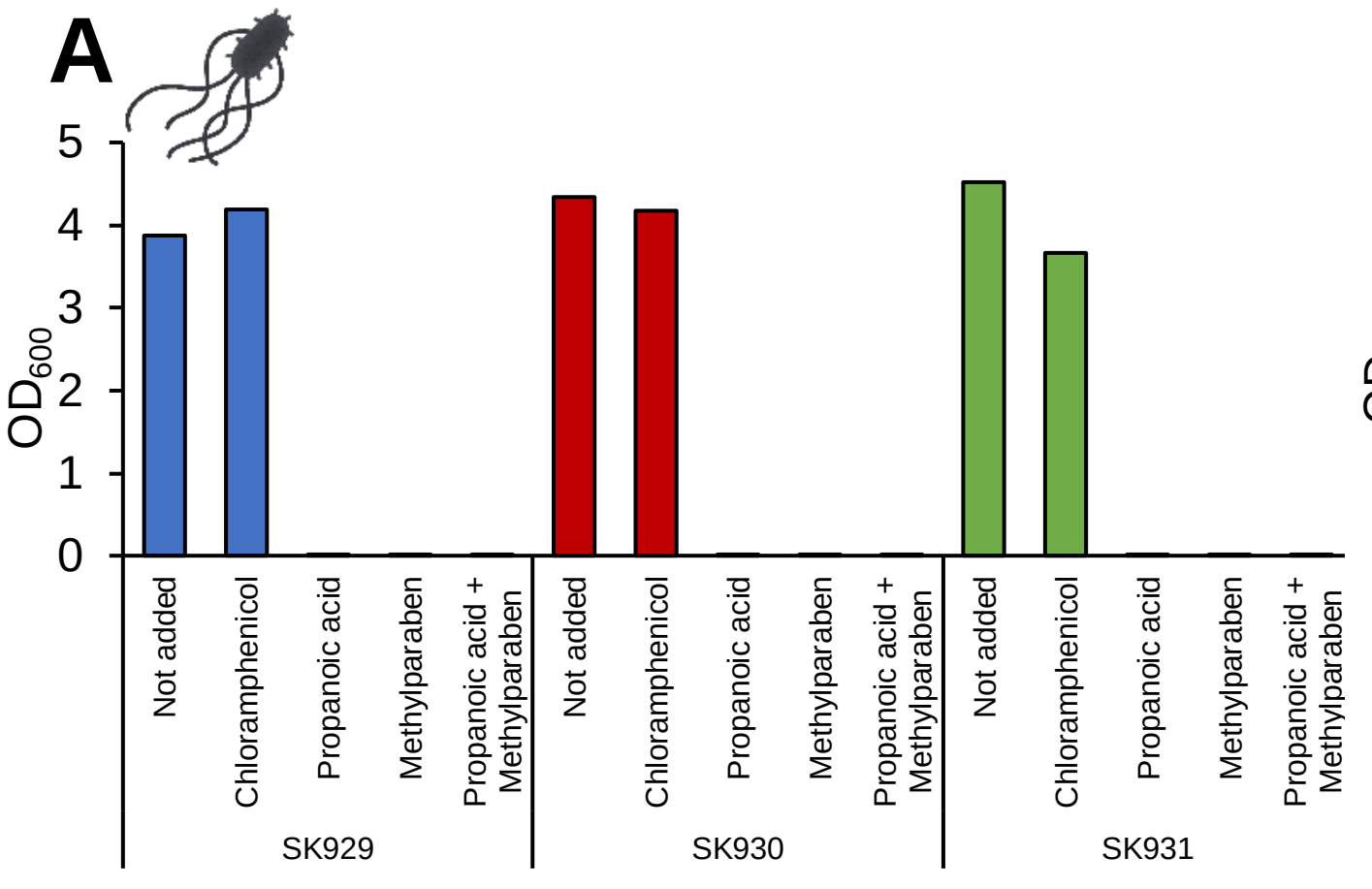

B

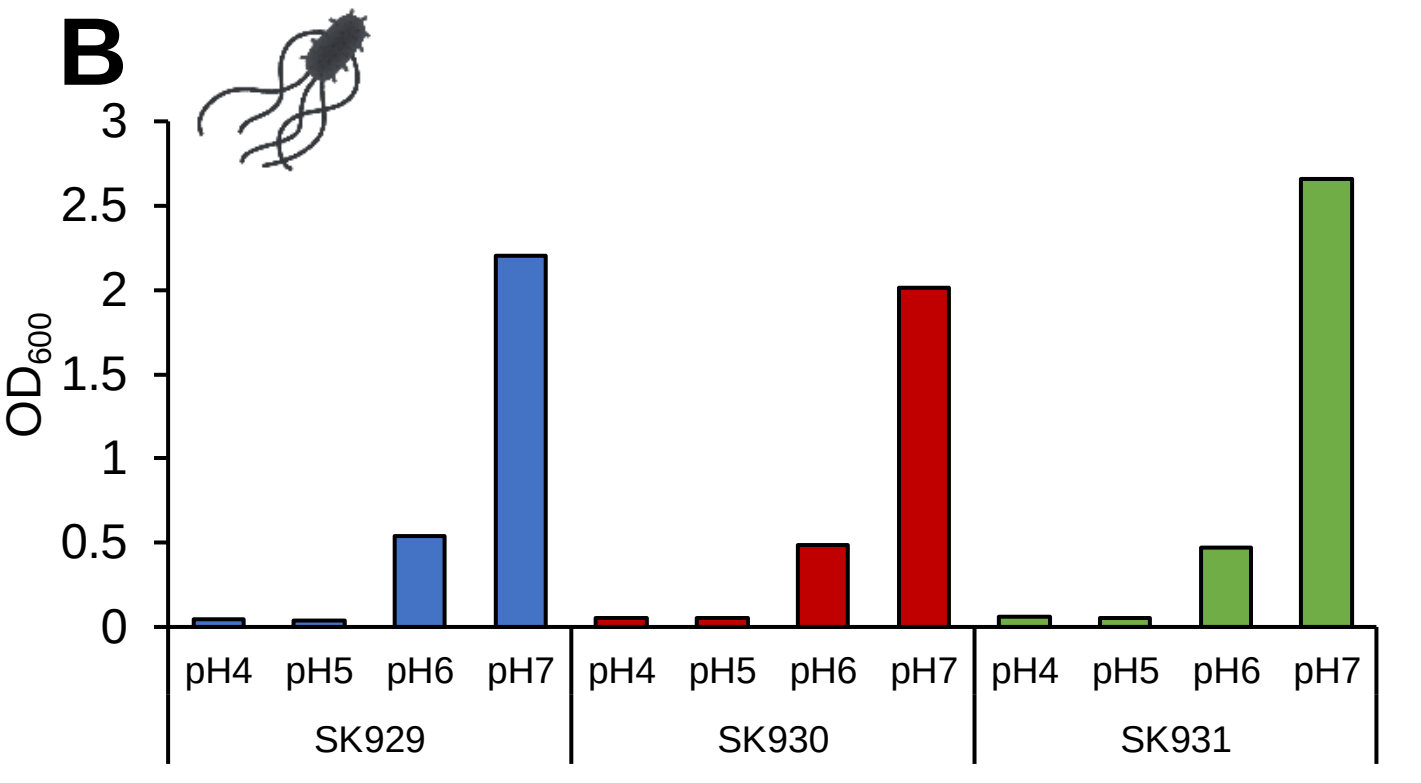

Extended Data Figure 3

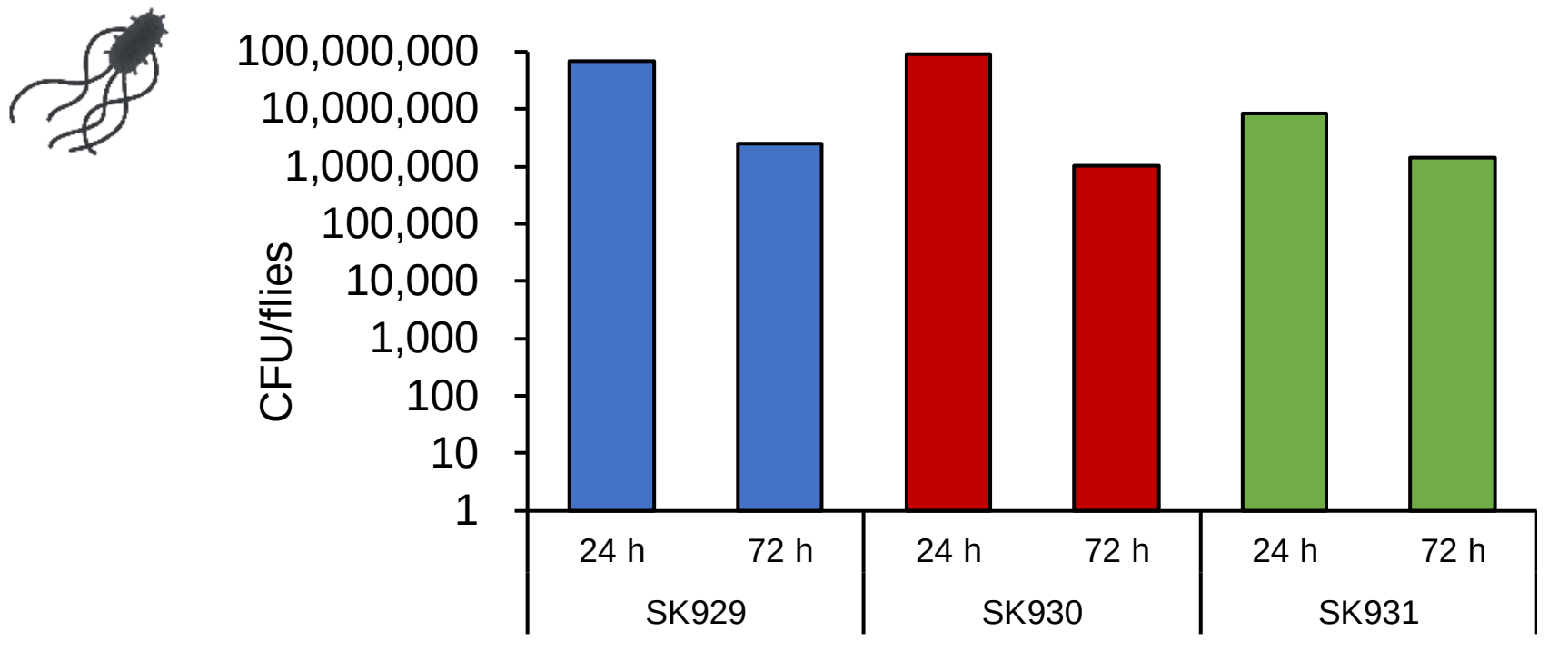

Supplement: Supplementary file 1 [file mbio.01484-26-s0001.pdf]
